# Changes in social participation between 1 and 2 years following moderate-severe traumatic brain injury

**DOI:** 10.3389/fresc.2022.945699

**Published:** 2022-07-22

**Authors:** Tessa Hart, Amanda Rabinowitz

**Affiliations:** Moss Rehabilitation Research Institute, Elkins Park, PA, United States

**Keywords:** traumatic brain injury, participation, social relations, outcomes, longitudinal studies

## Abstract

**Objective:**

To examine patterns of change in social participation in persons with moderate-severe traumatic brain injury (msTBI) between 1 and 2 years postinjury, and predictors of observed change.

**Participants:**

375 participants with msTBI enrolled in a single TBI Model System site.

**Measures and Methods:**

The dependent variable in a linear regression was a reliable change score for the Social Relations subscale of the Participation Assessment with Recombined Tools-Objective, administered at 1- and 2-year follow-ups. Predictors of change included demographics, injury severity, social and functional status at Year 1, and changes in function and life circumstances between years 1 and 2.

**Results:**

Social participation status did not change substantially for $\frac{3}{4}$ of the sample, while approximately equal proportions of the remainder improved or declined. The regression model was significant (*p* < 0.001). Improvement was predicted by private vs. public insurance and decline was predicted by a reduction in the FIM functional outcome measure from year 1 to year 2. Marginal predictors included education (higher levels predicting improvement) and year 1 marital status (single status predicting decline).

**Conclusions:**

Longitudinal change in social participation in the chronic phase of msTBI deserves further study, with attention to resource limitations and the impact of changes in functional status.

## Introduction

Participation is defined by the World Health Organization as a person's involvement in a life situation (1). Under this umbrella is typically included several broad domains of meaningful engagement in society: work, social relationships, and satisfying civic or avocational activities. The resumption of meaningful roles in society is a very high priority for people with moderate-severe traumatic brain injury (msTBI), but one that is difficult to attain. For example, decades of research attest to the low rate of return to competitive employment, and the science is advanced enough to support systematic reviews on the topic (2, 3).

Other aspects of participation have been studied less systematically, but are of equal importance both to people affected by msTBI and clinicians who treat them. Social participation, i.e., forming, resuming, and maintaining satisfying relationships with family and friends, and engaging in social activities, is a key contributor to emotional wellbeing and life satisfaction in this population (4, 5). There is increasing evidence, however, that this form of participation is negatively affected by msTBI. For example, one large-scale study showed that more than 40% of people with TBI reported long-term social isolation and loss of friendships (6). Other reports confirm that people with TBI report a decline in social participation relative to pre-injury levels (7, 8) and express a desire for more engagement in social and recreational activity (9). In a mixed-methods study of friendship in chronic TBI, nearly two-thirds of participants reported having *no* friends, and qualitative findings reflected a sense of “going downhill” with respect to social connections (5).

In order to develop successful treatment approaches to help affected people re-engage in social roles and activities, it is important to understand the natural history of postinjury changes in social participation. Particularly in light of recent evidence showing dynamic trends in recovery even years after msTBI (10, 11), it would be important to know whether, and in what ways, this form of participation changes over time in the chronic phase. It would also be of value to understand predictors of such change, to help identify individuals in need of treatment or prevention of unfavorable outcomes.

To date, there are few longitudinal investigations of social participation after the first year following msTBI. Using the Community Integration Questionnaire (CIQ) (12), which assesses the degree of independent participation in home, social, and productive activities, Willemse-van Son et al. (13) showed that social participation remained lower than pre-injury levels throughout the first 36 months postinjury. Ponsford and colleagues (6) found that problems with relationships and social activities actually increased over time, particularly for those with severe TBI. Predictors of change in overall community integration (including social integration) at several years postinjury include level of education, employment and marital status at time of injury, and severity of TBI (14).

At least one longitudinal study has been conducted using the Participation with Recombined Tools- Objective (PART-O), an instrument created within the Traumatic Brain Injury Model Systems (TBIMS) program. This tool was developed by synthesizing existing participation measures and validating the product in a large sample of persons with chronic msTBI (15). The PART-O was refined further using consumer input and empirical methods to derive a 17-item measure with 3 subscales: Productivity, Social Relations, and Out and About (i.e., community and recreational activity) (16). Erler et al. (17) examined the trajectory of PART-O scores over the first 5 years postinjury. While not focused on social relations specifically, this study reported that older age predicted both lower participation overall, and less recovery over time. Non-white race, depression, lower education, living alone, and worse cognitive and physical function at discharge from inpatient rehabilitation were all associated with worse participation overall, but appeared to have negligible impact on the rate or direction of change.

One gap in the social participation research to date is that previous longitudinal studies have examined changes and their predictors at the group level. While this approach contributes to our overall understanding of variables associated with participation, it is also important to examine different patterns of change among individuals. For example, some people may be at risk for declining participation over time, and their changes could be obscured by group-level analyses; yet understanding of the risk factors for decline could inform prevention efforts. Conversely, knowing more about the people who continue to improve their social lives within the chronic phase of msTBI might help us to promote positive change for others. There is likely to be heterogeneity in the patterns of change: for example, in a study focused on the relationship between changes in depression and changes in participation between years 1 and 2 postinjury, we found 6 classes of people distinguished by their joint trajectories on the two variables (18).

To begin to address these gaps, we performed a longitudinal study of people with chronic msTBI to: (1) examine descriptively the patterns of improvement, decline, and stability with respect to social participation between 1 and 2 years postinjury; and (2) determine the predictors of observed change. Due to the paucity of previous studies examining predictors of positive or negative change, we considered the study to be largely exploratory. We included non-modifiable predictors with potential relevance given previous literature (e.g., race, education, injury severity), but also examined potentially modifiable indicators of status at 1 year postinjury. In addition, we included, as predictors, changes in life circumstances or functional status between years 1 and 2 that might logically be associated with change in social participation.

## Methods

### Participants

Participants were 375 enrollees from a single center within the Traumatic Brain Injury Model Systems (TBIMS), a longitudinal study of TBI (19). Inclusion criteria stipulate age 16 or older, receipt of care in a TBIMS-affiliated trauma center within 72 h of injury, and direct transfer to an inpatient TBIMS rehabilitation unit. Enrollees have a penetrating or non-penetrating TBI with at least one of the following characteristics: Glasgow Coma Scale (GCS) score <13 on emergency admission (not due to intubation, sedation or intoxication); loss of consciousness >30 min (not due to sedation or intoxication); post-traumatic amnesia (PTA) >24 h; or trauma related intracranial abnormality on neuroimaging. Participants provide informed consent directly or by legal proxy. A standard data set is collected in acute care and inpatient rehabilitation and at scheduled intervals thereafter, including interviews near the 1- and 2-year anniversaries of the TBI.

To be included in this study, participants also had to have supplied complete data themselves (i.e., not by proxy) at both 1- and 2-year follow-ups on the outcome measure, the PART-O. Of note, cases were included only with follow-up interviews completed on or before 3/1/20, to obviate changes in participation brought on by the COVID pandemic.

#### Outcome measure

Changes in social participation between Years 1 and 2 was measured using the 17-item PART-O (15, 16), described earlier. This study focused on the Social Relations subscale, consisting of seven items asking about the presence and frequency of interpersonal interactions and intimate relationships. Examples of questions include “In a typical week, how many times do you… socialize with friends, in person or by phone; …give emotional support to other people; ….use the Internet for communication,” and “Not including your spouse or significant other, do you have a best friend in whom you confide?” Each item is scored from zero to five, and the subscale score represents the average of item scores.

To create a normally distributed score reflecting the degree and direction of change, we first calculated a change score (Year 2 – Year 1), and divided this by the Reliable Change Index (RCI) (20) denominator (0.49) supplied by a test-retest study conducted within the TBIMS (21). The resulting reliable change score was used as a continuous outcome variable in the regression analysis described below. For descriptive purposes we also classified participants into 3 groups: Improved, declined, or stayed the same relative to the RCI cutoffs. That is, change scores higher than 1.96 were classified as improved; those lower than −1.96 were classified as declined; and the remainder were considered unchanged within the standard error of measurement.

#### Baseline variables

Demographic data included age, sex, race/ ethnicity, and education (in years). Primary payer of medical services (public vs. private) was recorded as an additional marker of socioeconomic status (22). Severity of TBI was assessed using Time to Follow Commands (TFC), i.e., length of unconsciousness. This is measured in the TBIMS as the number of days between the date of injury and the first date that the injured person followed simple motor commands (i.e., GCS Motor = 6) twice within a 24-h period.

#### Year 1 variables

At the 1-year follow-up interview, the following variables were collected. Problematic substance use was coded yes/no according to TBIMS criteria, based on those of the Centers for Disease Control and Prevention (23) and the Substance Abuse and Mental Health Services Administration (24). Primary mode of transportation was coded as yes/no according to whether the participant drove a motorized vehicle at the time of follow-up. Functional status was measured with the self-report interview version of the FIM (25), which consists of 18 items rating the level of independence in self-care, mobility, communication, and cognition. Employment status was coded as productive (full-time student or competitively employed), unemployed, retired due to age, or other (volunteer, homemaker, etc.). Marital status was coded as single, married, or formerly married (separated, widowed, divorced). The variable “living with” was coded as alone, with spouse/significant other, with any other family, or with any other non-family. The PART-O Social Relations subscale score at 1-year follow-up was included as a covariate.

#### Changes between years 1 and 2

The following variables were coded as changed or unchanged from Year 1 to Year 2 follow-up: Employment status; marital status; person(s) living with. We reasoned that these types of changes might be associated with changes in social participation, but not in necessarily predictable directions. For example, leaving a job could result in either loss of social contact at the workplace, or freedom to socialize more. Therefore, we planned to conduct *post-hoc* descriptive analyses on any of the change variables that were significant predictors in the primary analysis.

For the FIM, we derived 3 ordinal groups (improved, unchanged, declined) using the same process as for the Social Relations subscale of the PART-O. That is, we divided the Year 2 – Year 1 change score by the RCI denominator supplied for the FIM by the TBIMS test-retest study cited above (21).

### Procedure

This project was approved and overseen by an Institutional Review Board and complied in full with the Helsinki Declaration. Demographic data and social history were obtained at baseline from medical records and participant/family interview. One- and 2-year follow-up data were collected by trained research assistants via telephone using a standard protocol, as close to the anniversaries of injury as feasible with a window of +/−8 weeks.

### Data analysis

Descriptive statistics were calculated for central tendency and variability for continuous measures and counts/percentages for nominal variables. Distributions of continuous predictors were examined for normality. TFC was log transformed to correct extreme positive skew. Categorical predictors were dummy coded as described in Measures; race/ethnicity was binary coded as white/nonwhite due to multicollinearity affecting the model when additional categories were included.

Linear regression was performed using jamovi (26), an open-source graphical user interface package that runs on the statistical computing language R (27). Predictors were entered using simultaneous entry of all variables in the model as a single block.

## Results

### Descriptive findings

Participant characteristics and baseline scores are displayed in Table 1. The sample was predominantly male, as is typical of msTBI, and quite racially diverse. Only about one-fifth were competitively employed or engaged in full-time study at 1 year postinjury. Fewer than one-third changed their employment or marital status, or the person(s) with whom they were living, from Year 1 to Year 2.

**Table 1:** Participant characteristics (*N* = 375).

***Baseline variables***		
Age (M/ SD)	42.3	18.9
Sex (no./% male)	271	72.3
Education (years; M/SD)	12.4	2.4
Race/ Ethnicity (no./%): White	191	50.9
Black	146	38.9
Hispanic	29	7.7
Other	9	2.4
Primary insurance (no./%): public	185	49.5
Private	189	50.5
Time to follow commands, days (M/ SD)	4.5	8.9
***Year 1 variables***		
Positive for problem substance use, no./%	74	19.7
Primary transportation, no./% drives motorized vehicle	81	21.6
FIM, total score (M/SD)	115.8	10.6
Employment status, no./%: competitively employed/FT student	82	21.9
Unemployed	140	37.3
Retired due to age	124	33.1
Other (volunteer, p/t student, homemaker)	29	7.7
Marital status, no./% single, never married	199	53.1
Married	82	21.9
Formerly married (widowed, separated, divorced)	94	25.1
Person(s) living with, no./%: Alone	49	13.1
With spouse or significant other	115	30.7
With other family	177	47.2
With other non-family	34	9.1
Depression (*n = 298*): No./ % minimal:	154	51.7
Mild	70	23.5
Moderate	38	12.8
Severe	36	12.1
***Variables showing change from Year 1 to Year 2***		
Employment status, any change: No./% changed	121	32.3
Marital status, any change: No./% changed	13	3.5
Person(s) living with, any change: No./% changed	102	27.2
FIM, total score: No./% improved	69	18.4
Stayed about the same	235	62.7
Declined	71	18.9

*M, Mean; SD, Standard deviation; FT, Full-time; PT, Part-time*.

Descriptive findings related to the outcome variable are displayed in Table 2. It may be seen that $\frac{3}{4}$ of the sample remained the same within the bounds of the RCI, while approximately equal proportions showed improvement and decline. Although there are no formal norms for the PART-O or its subscales, we note that both Year 1 and Year 2 scores for the overall sample are quite similar to those of a sample of persons with mixed disabilities (TBI, spinal cord injury, and stroke) reported in a study by Bogner and colleagues (mean 2.33, SD 0.9) (16). In comparison to a population-based sample of respondents without disability interviewed for the same study, the overall mean in our sample falls between the 5th and 25th percentile. All scores in Table 2 fall below the 50th percentile (score = 3.14) reported for the non-disabled participants in that study.

**Table 2:** Results for social relations subscale.

**Variable**	**Group defined by RCI**			**Whole sample**
	**Improved**	**Stayed same**	**Declined**	
	**(*n* = 49, 13%)**	**(*n* = 283, 75%)**	**(*n* = 43, 12%)**	
Subscale score year 1	1.54 (0.9)	2.21 (0.9)	2.85 (0.6)	2.19 (1.0)
Subscale score year 2	2.98 (0.9)	2.23 (0.9)	1.37 (0.7)	2.23 (1.0)
Year 2-year 1 change	1.45 (0.9)	0.02 (0.5)	−1.48 (0.5)	0.03 (0.9)

*Cells contain means with standard deviations in parentheses. Possible subscale scores are 0 – 5. RCI, Reliable change index*.

### Results of linear regression

The overall model accounted for 28.5% of the variance in reliable change in social participation [F(23/346)=5.99, *p* < 0.001)]. Greater positive change in social participation was associated with lower levels of social participation at Year 1 (*t* = −9.63, *p* < 0.001); private insurance (*t* = 2.03, *p* = 0.043); and FIM reliable change between Year 1 and Year 2, with those who were stable or improved on FIM exhibiting more positive change in social participation as compared to those who reliably declined on FIM (*t* = 2.17, *p* = 0.031 and *t* = 2.32; *p* = 0.021, respectively). There were marginally significant effects of years of education (*t* = 1.86; *p* = 0.063) and marital status at Year 1 (*t* = 1.85; *p* = 0.066) in the direction of greater positive change associated with more years of education, and married vs. single status at Year 1. See Table 3 for all regression coefficients.

**Table 3:** Linear regression model coefficients for predictors of change in social participation.

**Predictor**	**Estimate**	**SE**	***t***	***p***
Intercept	1.03	1.44	0.71	0.476
***Demographic characteristics***				
Age	−0.01	0.01	−1.74	0.084
Sex (male)	0.26	0.19	1.40	0.163
Race (white)	−0.01	0.18	−0.05	0.964
Rehab payor (private)	0.37	0.18	2.03	0.043
TFC (log transformed)	0.02	0.15	0.11	0.911
Education (years)	0.07	0.04	1.86	0.063
******Year 1 status******				
Driving status	−0.35	0.23	−1.51	0.133
Social participation	−1.11	0.12	−9.63	<0.001
Living with (reference: alone)				
With spouse/sig. other	0.25	0.36	0.69	0.489
With other family	−0.02	0.27	−0.01	0.994
With other non-family	0.47	0.37	1.28	0.200
Marital Status (reference: single)
Married	0.61	0.33	1.85	0.066
Formerly Married	0.00	0.23	0.01	0.989
Employment (reference: employed)				
Unemployed	−0.34	0.25	−1.33	0.185
Retired	−0.14	0.28	−0.49	0.624
Other	−0.13	0.38	−0.34	0.736
FIM	0.00	0.01	0.44	0.660
Problematic substance use	0.08	0.21	0.38	0.708
*Change between year 1 and year 2*
Living situation	−0.16	0.20	−0.76	0.445
Employment	0.24	0.20	1.18	0.237
FIM (Reference: declined)				
No change	0.47	0.22	2.17	0.031
Improved	0.68	0.30	2.3	0.021

## Discussion

We examined change, and predictors of change, in social participation between 1 and 2 years postinjury in a robust sample of persons with msTBI. One finding that might be viewed as positive was that for the majority of participants (75%), this type of participation did not change appreciably. On the other hand, comparison to scores from non-disabled participants (16) suggests that the level of social participation remained sub-optimal, even for those who improved between years 1 and 2.

Change in FIM and public vs. private insurance emerged as significant predictors of change in social participation. The association with FIM was in the expected direction and indicates that a decline in physical or cognitive status could also signal a reduction in social contact. While not surprising, this result could be useful for clinicians to anticipate and foster different social opportunities, or compensatory strategies for continuation of participation, for those with declining functional status. Interestingly, the FIM declines were apparently not age-related, as the age distributions in the three participation change groups were nearly identical (data not shown). Age had a slight negative impact in the regression model, but this did not reach significance (see Table 3). In a previous study using the PART-O, older participants showed less improvement in participation over time (17). Comparison of our findings to previous literature is otherwise difficult, as most studies have examined predictors of overall participation as opposed to change in specific activities. Education, which has been associated with better postinjury participation (14, 17), was marginally significant in our model but in the direction noted in prior work. Being single at year 1 also approached significance in our model as a predictor of decline in social participation compared to being married, presumably because marriage confers a richer network of friends and social contacts that help to guard against loss of social activity.

In contrast to at least one study of predictors of overall community integration (14), the initial severity of TBI did not emerge as a significant predictor in the present investigation. While negative findings must be interpreted with caution, it is reasonable to believe that the impact of the neurologic insult on outcomes in the chronic phase may be less salient as other factors exert more influence on recovery. For example, patients may have identical durations of loss of consciousness (the severity index used in this study) yet experience very different levels of subacute and post-acute treatment, community resources, and other factors that affect the level of social participation and other forms of role resumption. In this vein, the significant contribution of the insurance variable (an indirect measure of socioeconomic status) to the present findings underscores the importance of considering resource limitations in the maintenance of social participation in the chronic phase of msTBI. Future research should explore more directly the impact of financial factors, as well as other resources such as family support.

Another fruitful direction for future research could involve alternative, and perhaps richer, methods for measuring social participation. While the PART-O used in this study has the advantage of brevity and has been well validated for use in msTBI, the items focus on frequency of specific activities or presence/absence of close friends and romantic partners. This does not allow assessment of the respondents' satisfaction with his/her level of participation or desired changes in patterns of activity. More recently, the Traumatic Brain Injury Quality of Life (TBI-QOL) computer-adaptive measurement system has included development of separate scales for assessing the ability to participate in social roles/activities, and one's satisfaction with those roles/activities (28). Other researchers have supplemented assessment of quantitative (e.g., frequency-based) participation with assessment of qualitative factors such as one's sense of enfranchisement, or inclusion in one's community (29). We encourage continued efforts to include the perspectives of affected individuals in the assessment of social relations and other forms of societal participation.

Limitations of this study should be noted when interpreting the results. When using any longitudinal database, one is constrained by the available data elements. Thus, there may be variables with a strong connection to change in social participation that are not captured in the TBIMS dataset. For example, with the data available to us, we cannot illuminate the cause(s) of functional decline observed in a portion of our sample. Although our regression model was significant overall, the modest amount of variance accounted for suggests that further study with different variables would help to explain changes in the chronic phases of TBI. In addition, our sample was limited to people who were able to provide self-report data, and thus may not apply to more severely impaired individuals. Nor do our findings necessarily apply to those with mild TBI, who are not represented in the TBIMS project.

In conclusion, changes in social participation in the chronic phase of recovery from msTBI deserve more attention. The potential impacts of resource limitations and deleterious changes in functional status should be examined more closely in future research.

## Data availability statement

The data analyzed in this study is subject to the following licenses/restrictions: Data must be requested from the TBI Model System National Data Center. Requests to access these datasets should be directed to https://www.tbindsc.org.

## Ethics statement

The studies involving human participants were reviewed and approved by Institutional Review Board, Einstein Healthcare Network. Written informed consent to participate in this study was provided by the participants or by participants' Legally Authorized Representative.

## Author contributions

Conception and design of work, acquisition, analysis, interpretation of data, drafting and revising the work for intellectual content, and final approval of submitted work: TH and AR. Both authors agree to be accountable for all aspects of the accuracy and integrity of the final work. Both authors contributed to the article and approved the submitted version.

## Funding

The contents of this manuscript were developed under a grant from the National Institute on Disability, Independent Living, and Rehabilitation Research (NIDILRR grant number 90DPTB0004). NIDILRR is a Center within the Administration for Community Living (ACL), Department of Health and Human Services (HHS). The contents of this manuscript do not necessarily represent the policy of NIDILRR, ACL, or HHS, and you should not assume endorsement by the Federal Government.

## Conflict of interest

The authors declare that the research was conducted in the absence of any commercial or financial relationships that could be construed as a potential conflict of interest.

## Publisher's note

All claims expressed in this article are solely those of the authors and do not necessarily represent those of their affiliated organizations, or those of the publisher, the editors and the reviewers. Any product that may be evaluated in this article, or claim that may be made by its manufacturer, is not guaranteed or endorsed by the publisher.
